# RNA-Seq Characterization of Spinal Cord Injury Transcriptome in Acute/Subacute Phases: A Resource for Understanding the Pathology at the Systems Level

**DOI:** 10.1371/journal.pone.0072567

**Published:** 2013-08-09

**Authors:** Kenian Chen, Shuyun Deng, Hezuo Lu, Yiyan Zheng, Guodong Yang, Dong Kim, Qilin Cao, Jia Qian Wu

**Affiliations:** 1 The Vivian L. Smith Department of Neurosurgery, University of Texas Medical School at Houston, Houston, Texas, United States of America; 2 Center for Stem Cell and Regenerative Medicine, UT Brown Institution of Molecular Medicine, Houston, Texas, United States of America; University of Louisville, United States of America

## Abstract

Spinal cord injury (SCI) is a devastating neurological disease without effective treatment. To generate a comprehensive view of the mechanisms involved in SCI pathology, we applied RNA-Sequencing (RNA-Seq) technology to characterize the temporal changes in global gene expression after contusive SCI in mice. We sequenced tissue samples from acute and subacute phases (2 days and 7 days after injury) and systematically characterized the transcriptomes with the goal of identifying pathways and genes critical in SCI pathology. The top enriched functional categories include “inflammation response,” “neurological disease,” “cell death and survival” and “nervous system development.” The top enriched pathways include LXR/RXR Activation and Atherosclerosis Signaling, etc. Furthermore, we developed a systems-based analysis framework in order to identify key determinants in the global gene networks of the acute and sub-acute phases. Some candidate genes that we identified have been shown to play important roles in SCI, which demonstrates the validity of our approach. There are also many genes whose functions in SCI have not been well studied and can be further investigated by future experiments. We have also incorporated pharmacogenomic information into our analyses. Among the genes identified, the ones with existing drug information can be readily tested in SCI animal models. Therefore, in this study we have described an example of how global gene profiling can be translated to identifying genes of interest for functional tests in the future and generating new hypotheses. Additionally, the RNA-Seq enables splicing isoform identification and the estimation of expression levels, thus providing useful information for increasing the specificity of drug design and reducing potential side effect. In summary, these results provide a valuable reference data resource for a better understanding of the SCI process in the acute and sub-acute phases.

## Introduction

Spinal cord injury (SCI) is one of the most debilitating neurological diseases. About 300,000 people are currently living with SCI in the United States and nearly 11,000 new cases are reported annually [1]. The expense associated with the medical care is very high, but clinically available treatments provide only modest benefit [2,3]. At present, there are no effective treatments for this devastating neurological disorder.

Previous studies indicated that in the acute phase of SCI, primary damage occurs as a direct result of trauma when resilience thresholds are surpassed, leading to immediate physical, biochemical and cellular alterations. Primary injury triggers multiple secondary injury cascades that cause further tissue loss and dysfunction [4]. Unraveling the detailed molecular events and especially the key genes and pathways would shed light on both understanding the injury mechanisms and developing therapeutic strategies.

Due to technical limitations, previous studies have usually focused on a small number of genes and pathways at a time [5,6,7,8,9,10,11,12,13,14,15], and thus did not provide a comprehensive view of the complex mechanisms contributing to SCI pathology. Considering the large number of biochemical cascades and cellular reactions involved, a global analysis is necessary in order to elucidate the pathophysiology of SCI. Although the use of cDNA microarray and genechip technologies have provided valuable insights into the disorder during the last decade [16,17,18,19,20,21], microarray technology suffers from limitations in resolution, dynamic range and accuracy [22]. Recent advances in Next Generation Sequencing technology, specifically RNA-Seq technology, make it possible to globally map transcribed regions and quantitatively analyze RNA isoforms at an unprecedented level of sensitivity and specificity [23,24].

Notably, recent large scale RNA-Seq studies have revealed that most mammalian genes generate multiple RNA transcripts through alternative splicing [25]. Alternative transcripts encoding proteins with distinct functions or regulatory properties can have profound physiological effects. Since the formation of these gene isoforms is tissue and context-dependent, targeting specific isoforms has the potential to improve drug efficacy and reduce side effects [26,27]. Herein, we use RNA-Seq technology to investigate the isoform expression of genes during the SCI process, thus revealing another layer of information that is valuable for targeted therapy and drug design.

In this study, we have generated a comprehensive reference data resource for a better understanding of the SCI process in the acute and sub-acute phases. Comparing to the microarray data from a prior SCI study that is similar to our study, RNA-Seq data showed higher sensitivity, larger dynamic range and was able to identify more differentially expressed genes. Importantly, we have investigated the SCI process as a whole, rather than as a collection of single gene entities. Based on the data generated, we characterized the functions and pathways involved, and developed a systems biology based framework to analyze the SCI global gene networks that will enable the identification of potential key determinants in the acute and sub-acute phases. Some candidate genes that we identified have been shown to play important roles in SCI, thus supporting the validity of our approach. There are also many additional genes that are identified whose functions in SCI have not been well studied. In the future, we will examine the roles of these interesting genes using animal models and functional tests.

## Methods

### Animal model and tissue preparation

All animal care and surgical interventions were undertaken in strict accordance with the Public Health Service Policy on Humane Care and Use of Laboratory Animals, Guide for the Care and Use of Laboratory Animals, and with the approval of Animal Welfare Committee at the University of Texas Health Science Center at Houston.

A total of 24 female C57BI/6J mice (10-16 weeks of age; 20-25g; The Jackson Laboratory, Bar Harbor, ME) were used with 3 mice pooled together in each biological replicate: sham control (n=2), 2 and 7 days after SCI (n=3). The surgical procedure for SCI were described previously [28]. Briefly, after anesthetization with a mixed solution of ketamine (80 mg/kg ip) and xylazine (10 mg/kg ip), mice received a dorsal laminectomy at the 9^th^ thoracic vertebral (T9) level to expose the spinal cord and then a moderate T9 contusive injury using an Infinite Horizons impactor (Precision Systems and Instrumentation) at 60 kdyn with the spine stabilized using steel stabilizers inserted under the transverse processes one vertebra above and below the injury [29]. The sham control mice received only a dorsal laminectomy without contusive injury. Afterwards, the wound was sutured in layers, bacitracin ointment (Qualitest Pharmaceuticals, Huntsville, AL) was applied to the wound area, 0.1mL of a 20 mg/ml stock of gentamicin (Butler Schein, Dublin, OH) was injected subcutaneously, and the animals recovered on a water-circulating heating pad. Then mice received analgesic agent, buprenorphine(0.05 mg/kg, SQ; Reckitt Benckise, Hull, England) twice a day for two days. Bladders were manually expressed until automatic voiding returned spontaneously, which generally was within 7 days. The mice locomotion tests, Basso Mouse Score, were performed at 2 and 7 days post-injury before collecting the injured spinal cord tissues to confirm the injury severity in each mouse is consistent with moderate contusion SCI. At 2 or 7 days after SCI, the mice were anesthetized again with ketamine and xylazine and perfused briefly with normal physical saline. The injured spinal cords were then dissected. 0.5 mm pieces of spinal cord were cut in the injured epicenter and frozen in liquid nitrogen and processed for RNA isolation. Histological staining was done by the iron-eriochrome cyanine R (EC) staining. The spinal cords from the epicenter, 6mm and 12mm to the epicenter caudally and rostrally were used for staining.

### RNA-Sequencing

RNA-Seq was performed on the polyadenylated fraction of RNA isolated from tissue samples of acute (2D) and subacute phase (7D) and normal tissues (control, denoted as CTR hereafter). Three biological replicates were used for 2D and 7D, and two biological replicates were used for sham control. Trizol was used to extract total RNA and the quality was accessed by Bioanalyzer. Only samples with RIN>8 were used for library construction. 150-300 ng total RNAs were used for each sequencing library. RNA samples were polyA selected and paired-end sequencing libraries were constructed using TruSeq RNA Sample Prep Kit as described in the TruSeq RNA Sample Preparation V2 Guide (Illumina). The samples were then sequenced using the Illumina HiSeq sequencer.

### Mapping sequence reads to the mouse genome

The 100bp paired-end RNA-Seq reads were mapped to the mouse reference genome (version mm9) by the Top hat software [30] (version 1.3.3) which uses the Bow tie read mapper [31] (version 0.12.7). Because we used spinal cord tissue which includes mixed cell types, in order to reconstruct reliable gene structures and obtain accurate expression level estimations, Top hat was fed with the option of “–no-novel-juncs”. Only reads compatible with the annotated gene were considered. The final step in setting up the parameters was to designate option -G, a prompt which supplies Top hat with known gene model annotations [32].

### Transcriptome reconstruction and expression level estimation

Mapped reads were assembled into RNA transcripts using the Cufflinks software [33] (version 1.3.0). Cufflinks was run using the annotation file (downloaded from Cufflinks web site) of known genes and the mapped reads produced by Top hat. Fragments Per Kilobase of exon model per Million mapped fragments (FPKM) value (a normalized gene expression value that are comparable between different samples and genes) together with confidence intervals were estimated for each replicate. The whole process (from mapping to FPKM generation) was automated using our in-house pipeline.

### FPKM threshold determination

**Figure 1:**
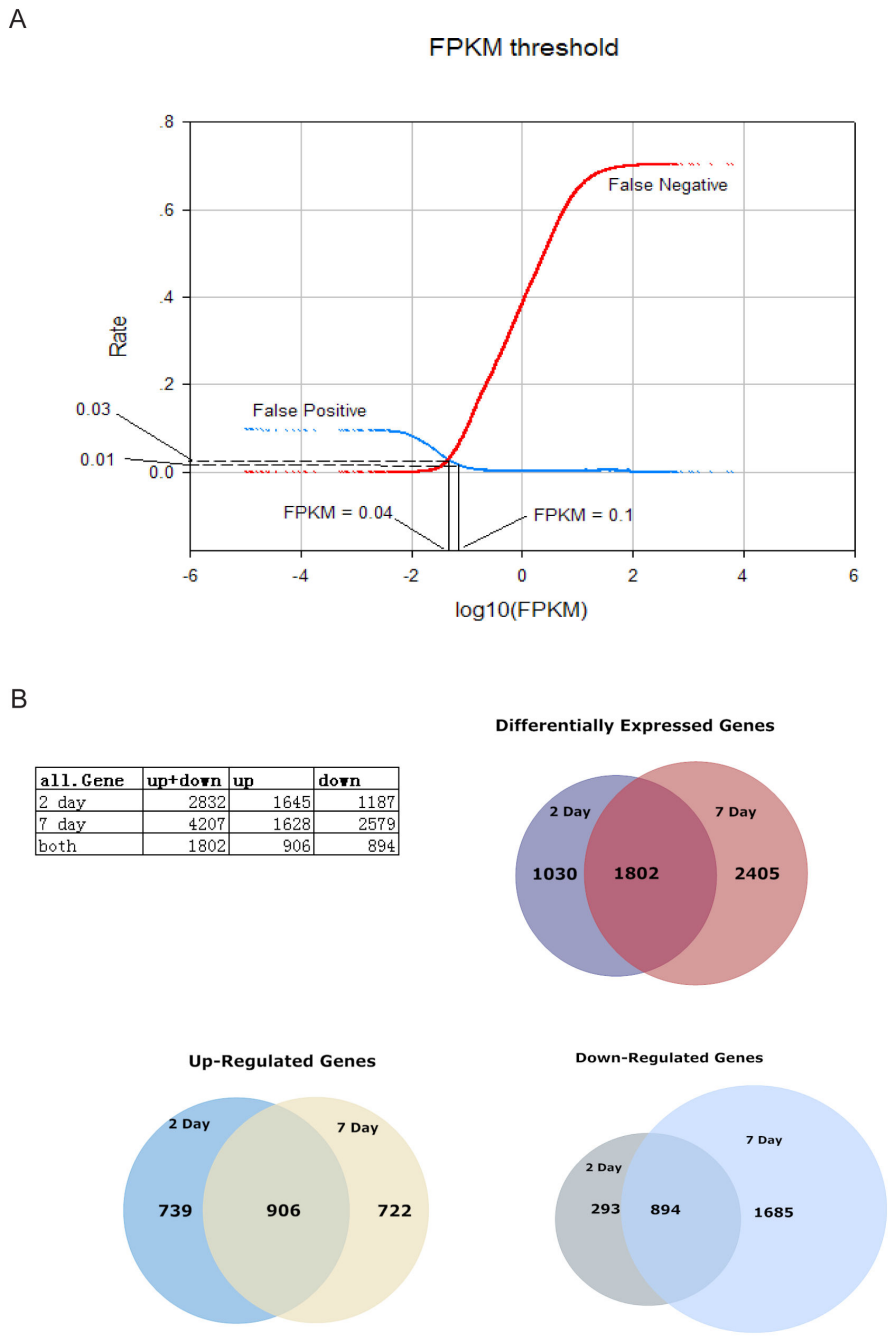
Identification of differentially expressed genes. (A) Detection threshold determination. False positive and negative rates for the detection of genes as a function of detection threshold used, demonstrating how a detection threshold of 0.04 FPKM was determined. A more conservative threshold 0.1 FPKM was chosen for downstream analysis (the probability that a transcript can be reliably detected is ~0.99). (B) Table and Venn diagrams show the distribution of genes that changed > 2 fold and were statistically significant (t-test p < 0.05) in 2D and 7D stages.

To facilitate downstream analyses (such as gene expression fold change analysis etc.) and to access the accuracy and reliability of our RNA-Seq experiments, we conducted an analysis based on the 95% confidence intervals of FPKM values calculated by Cufflinks. For each gene, if its estimated FPKM value had a lower confidence bound of 0 then it was labeled as “unreliable” (had a possibility of being 0); in contrast, if it had a lower confidence bound larger than 0, then it was labeled as “reliable”. The numbers of “reliable” and “unreliable” FPKM values were counted at each FPKM level. False positive and false negative rate curves were produced in order to identify a FPKM value that is optimized for both false positive rate and false negative rate (Figure 1A). According to the above procedure, we identified that at ~0.04 FPKM level, the probability that a transcript can be reliably detected is ~0.97. At 0.1 FPKM level, the probability that a transcript can be reliably detected is ~0.99. To be conservative and for the convenience of calculating fold change, we choose 0.1 FPKM as the threshold for following analysis. Any FPKM that is below 0.1 were set to 0.1 when calculating fold change of different samples. This is similar in microarray data analysis, the background intensities below threshold (floor value) are often set to the threshold to avoid ratio inflation [34].

### Clustering analysis

To group genes according to their expression patterns during the SCI process, c-means clustering of genes expression across CTR, 2D and 7D stages was performed using R package mfuzz (version 2.16.1) [35]. Only genes that have fold change > 2 between at least 2 stages were used for the clustering (7239 genes). To evaluate the consistency of expression estimation among samples, unsupervised hierarchical clustering was performed using the hclust method with complete linkage in R (Pearson correlation metric). Top 3000 genes with highest variation across SCI stages were used.

### Comparative analysis of RNA-Seq and prior microarray SCI study

Microarray gene expression data of a similar SCI study was downloaded from GEO (Gene Expression Omnibus http://www.ncbi.nlm.nih.gov/geo/query/acc.cgi?acc=GSE5296). Briefly, global changes in gene expression in C57BL6 mouse model of contusion injury were evaluated using Affymetrix Mouse Genome 430 2.0 arrays. Mice were subjected to a moderate injury at the T8 spinal segment under isoflurane anesthesia. Sections of spinal cord were analyzed from the site of impact at 0.5, 4, 24, 72 h and 7 and 28 days after injury (n=3 per group), sham-injury (n=2 per group), or naive mice (n=2). Four mice were pooled for each individual n, for a total of 12 mice for each injury time point. The time points that are in common with our RNA-Seq data (sham control (0 hour) and 7 days after injury) were used for comparison analysis. We downloaded the. cel files from GEO and processed those using Bioconductor R packages (version 2.12). Intensity data were normalized per chip using MAS5 algorithm implemented through Bioconductor affy package. Gene symbols and gene names were downloaded from Affymetrix NetAffx Mouse430_2 annotations file (http://www.affymetrix.com/support/technical/byproduct.affx?product=moe430-20).

All probe sets except the AFFX control probe sets were then mapped to gene identifiers using above mentioned annotation file. In the situation when multiple probe sets were mapped to one gene, the probe set with the greatest standard deviation of expression values was selected.

To evaluate data correlation between microarray intensities and RNA-Seq FPKM values, spearman correlation were calculated. Only those genes that are detected on both platforms were used for calculation. In order to compare the ability of detecting differential expressed (DE) gene by microarray and RNA-Seq, fold-change (in log scale) was calculated using probe intensities of microarray or FPKM values of RNA-Seq. Differential expressed genes were determined as fold-change > 2 and p < 0.05 using an unpaired two-sided t-test.

### Quantitative RT-PCR

Quantitative RT-PCR was performed to validate gene expression changes of C3AR1, CCL7, CD22, CD36, CLEC6A, FCER1G, FCGR2B, Il7r, LPL, MSR1, and PTX3. Total RNAs from injured and CTR mice were purified with Trizol reagent (Invitrogen) and DNA was removed by DNase treatment (Invitrogen). First-strand cDNA was synthesized from 900 ng total RNA in a 20-µl volume using SuperScript® Double-Stranded cDNA Synthesis Kit and SuperScriptII (Invitrogen). The PCR reaction consisted of 1µl of 1:10-diluted cDNA, 5 µl of iTaq SYBR Green Supermix With ROX (Bio-Rad Laboratories, Inc.) and 100 pmol each of 5’ and 3’ primers (shown as below) in a total volume of 10 µl and was performed in ABI 7900HT (Applied Biosystems) for 40 cycles with denaturation at 95° C for 15 seconds, annealing at 60° C for 30 seconds and extension at 72° C for 30 seconds. RNA without reverse transcriptase treatment was used as negative control. All qPCR reactions were performed in triplicate and Ct values were averaged. Relative Expression Fold Change was calculated using 2^-ΔΔCt^ method [36]. The calculation was performed between 2D/CTR and 7D/CTR respectively. Housekeeping gene Hprt was used. Amplification primers for qRT-PCR were designed by using PrimerExpress3.0 Software (Applied Biosystems, Life Technologies). The primer sequences were:

C3AR1 forward primer: TCTCACCCTGGCCGATTTC, C3AR1 reverse primer: GGGATAAGTTTGCACAGGAACAA;

CCL7 forward primer: AATGTGCCTGAACAGAAACCAA, CCL7 reverse primer: CTTAGGACCGTGATCAACACATTT; CD22 forward primer: CAGGGACCCCAGCAACAC, CD22 reverse primer: ATTCACATTCTCATAATCCCCATAGG; CD36 forward primer: CCTGGGAGTTGGCGAGAAA, CD36 reverse primer: CCAATAACAGCTCCAGCAATGA, CLEC6A forward primer: TCTGTGGCATTTAACTCAAGTGTGT, CLEC6A reverse primer: TTGCATCTGTCTCCAAAATACGA; FCER1G forward primer: CCCGGAGCCAGGAGACATAT, FCER1G reverse primer: GAATTAGAAGTGGGAAAAGAATGCA; FCGR2B forward primer: TCCAGGTGCTCAAGGAAGACA, FCGR2B reverse primer: CGGATGGACCTCCCATTGT; IL7R forward primer: CACAAGAACAACAATCCCACAGA, IL7R reverse primer: ACTCGCTCCAGAAGCCTTTG; LPL forward primer: CGCTCCATTCATCTCTTCATTG, LPL reverse primer: AGGCAGAGCCCTTTCTCAAAG; MSR1 forward primer: TTTACCAGCAATGACAAAAGAGATG, MSR1 reverse primer: AAGGGATGCTGTCATTGAACGT; PTX3 forward primer: GGACAACGAAATAGACAATGGACTT, PTX3 reverse primer: CGAGTTCTCCAGCATGATGAAC; One internal control: HPRT (forward primer: TATGCCGAGGATTTGGAAAA, reverse primer: ACAGAGGGCCACAATGTGAT).

### Systematic identification of potentially important genes in SCI acute/subacute phases

In order to identify genes that play important roles during SCI, we employed a systems based approach. First, differentially expressed genes were selected for network construction. With the purpose to create a reasonable size network that included an essential number of genes involved in SCI process, we selected top 10% up/down-regulated genes (fold change > 2) for the network construction. In the second step, selected differentially expressed genes were uploaded into ingenuity pathway analysis. Networks for acute (2D) and subacute (7D) phases were constructed using IPA software, and merged into a global network for each stage. Genes that do not have connections with other networks were left out. Briefly, the IPA software constructs networks that are optimized for both interconnectivity and the number of focus genes (genes in the uploaded dataset). From these focus genes, the network grows using neighborhood genes. Thus, some genes that are not in the focus gene dataset are also included in the final network (the white nodes). In 7D, because there are many more down-regulated genes than in 2D, we constructed separate networks using up- or down-regulated genes. In the third step, the connection number of the genes that appeared in the network was obtained from IPA and used as an indicator of the genes’ involvement in the constructed network. In the fourth step, genes were further selected based on the following criteria: location on plasma membrane or in extra cellular space and fold-change > 2. Because there are a relatively small number of genes that have drug information in ingenuity knowledge base, we also included the genes with available drug information regardless of their cellular location. We then calculated an index number considering both the fold change and the connection number of a gene in the constructed network and ranked the genes based on this number (relevance index: RI = abs(log(Fold change)) *(Connection number)). We searched related literatures in the PubMed database to further select interesting genes for qRT-PCR validation.

### Gene set enrichment analysis

Gene set enrichment analysis (GSEA: http://www.broad.mit.edu/GSEA) [37] was used to understand the biological pathways involved in SCI. We used the built-in C2 and C5 curated gene sets (Molecular Signatures Database (MSigDB 3.0: www.broadinstitute.org/gsea/msigdb)). The statistical significance of GSEA was analyzed using 1000 permutations. Enrichment was compared among Normal, 2D SCI and 7D SCI stages in this study. A positive enrichment score indicates that the specific molecular signature correlated with the SCI phenotype. The significantly enriched gene sets, such as those related to hypoxia and energy metabolism, were further analyzed. Briefly, the genes in the leading subset (which mainly contribute to the enrichment score) were analyzed using IPA in order to study their functions and networks.

## Results

### Transcriptome dynamics at acute/subacute phases of SCI

Clinically relevant contusion SCI model was used in this study. The contusion SCI resulted in a central injury devoid of neurons and glias due to the loss of neural tissue and an outer rim of spared white matter in the injury epicenter (Figure S1). The central injury gradually increased while the peripheral spared white matter gradually decreased in the caudal and rostral spinal cords (Figure S1). The injury size and the percentage of spared white matter were closely correlated with the injury severity determined by the injury forces of the contusion impactor [38,39]. Moderate contusion SCI was used in the present study and the injury severity was confirmed by the histology staining and the locomotion test: Basso Mouse Score.

Transcriptomes were reconstructed using our in-house pipeline (see details in Materials and Methods). ~95% of all reads were mapped to the mouse reference genome (NCBI37/mm9) and over 100 million reads were recognized as spanning a splice junction (Table S1) (all sequences have been submitted to GEO database: GEO Series accession number GSE45376). The estimated normalized expression levels were reported in FPKM. To achieve accurate estimation of expression level, we focused on known transcripts in our study (Table S2). We identified a statistical threshold for the reliability of expression measurement based on FPKM values (see Materials and Methods). False positive and false negative rate curves for each biological replicates were then generated to identify an optimized FPKM threshold that minimized both false positive rates and false negative rates (Figure 1A). These analyses reveal that when a FPKM value is > ~0.04, the reliability of the FPKM is estimated to be ~0.97; when a FPKM value is > ~0.1, the reliability of the FPKM ~0.99, reflecting a high quality of expression level estimation. Therefore, we set the threshold to 0.1. FPKM values below 0.1 were set to 0.1 as the lowest expression value to avoid ratio inflation when calculating fold change of gene expression. Pearson correlation coefficients of global gene expression between replicates were high (r > 0.94) (Figure S2 A), indicate high consistency between replicates. Unsupervised hierarchical clustering of RNA-Seq data also shows good correlations among replicates (Figure S2 B).

Quantitative comparison of 2D and 7D expression to CTR expression allowed for the identification of all genes that were differentially expressed in these two stages. All genes up/down-regulated by more than 2-fold and statistically significant (t-test p < 0.05) were counted and summarized in table (Figure 1B Venn diagram). According to our criteria, there were 2832 differentially expressed genes (DE genes) at 2D stage, and 4207 DE genes at 7D stage. 1802 genes differentially expressed in both 2D and 7D stages. 7D had more DE genes compared to 2D, because there were more down-regulated genes in 7D than in 2D. 1187 genes were down-regulated in 2D; while 2579 genes were down-regulated in 7D. Down-regulated gene number increased in 7D samples because of increased neural cell death. Among these, 894 genes were significantly down-regulated in both 2D and 7D. Genes involved in ‘Nervous System Development and Function’ were enriched. There were similar numbers of genes up-regulated in 2D and 7D. 1645 genes were up-regulated in 2D, and 1628 genes were up-regulated in 7D. A large number (906) were up-regulated in both 2D and 7D, involving more than1/2 up-regulated genes in 2D or 7D. Genes related with ‘Inflammatory Response’ were enriched.

We used cluster analysis (c-means clustering) to separate 7239 genes that changed >2 folds over the time course into 9 groups (Figure S2 C). ‘Nervous system development and function’ was among the top enriched functional categories in clusters 3, 4 and 8, while ‘inflammatory response’ was among the top enriched functional categories in clusters 1, 2, 5 and 9.

### RNA-Seq in comparison with microarray study

A number of studies comparing the RNA-Seq and microarray accuracy, dynamic range and differential expression detection showed that RNA-Seq is superior to microarray and can provide novel information [22,40,41,42,43,44]. To evaluate the strength and validity of our RNA-Seq data, we searched GEO for microarray experiments that are comparable to our study in terms of the animal model and the type of injury. The only comparable microarray SCI experiment is GSE5296 (see Material and Method). In this study, C57BL6 mouse model of contusion injury (at thoracic vertebral (T8) level) were evaluated using Affymetrix Mouse Genome 430 2.0 arrays. Global changes in gene expression were monitored at different time points (sham control, 0.5, 4, 24, 72 h and 7 and 28 days after injury). We compared the common time points of the microarray study with our data at 0 hour (sham control) and 7 days after SCI. The spearman correlations between 0 hour (sham control) and 7 days after SCI are very high in the microarray study and are close to the correlations between biological replicates of the same time point (Figure S3 A). RNA-Seq shows increased detection of differential expressed genes between CTR and 7D (Figure S3 B). Together, our analysis results demonstrated that RNA-Seq is more sensitive and has broader dynamic range than microarray (Figure S3 B,C).

### Enriched functional categories and pathway analysis

The large number of DE genes in 2D and 7D demonstrates a dramatic impact of the injury to the transcriptome. To systematically characterize the functions of the DE genes, we searched for the enriched functional categories among genes that changed most significantly. To balance the numbers of up- and down-regulated genes selected, top 10% up-regulated and down-regulated genes (genes fold change >2) were used for the analysis. Top enriched functional categories included ‘inflammation response’, ‘neurological disease’, ‘cell death and survival’ and ‘nervous system development and function’ at both 2D and 7D. However, the specific genes in these functional categories and gene numbers varied between 2D and 7D (Table S3). The number of genes involved in the functional categories ‘inflammation response’, ‘cell death and survival’ and ‘nervous system development’ all increased in 7D (101, 73 and 111, respectively) compared with 2D (77, 47 and 48, respectively), reflecting that inflammation, cell death, and nervous system necrosis become more extensive in 7D than in 2D samples. Most genes involved in ‘nervous system development’ were down-regulated. In 2D, some genes, such as Tnf, Il6, Lif, Il1b, Adipoq, Il1rn and Ccl13 were involved in multiple functional categories, indicating that they may play multiple roles in SCI acute phase. In 7D, CXCR4, Grin1, Spp1, Cybb and Ccl13 were also involved in multiple functional categories.

**Figure 2:**
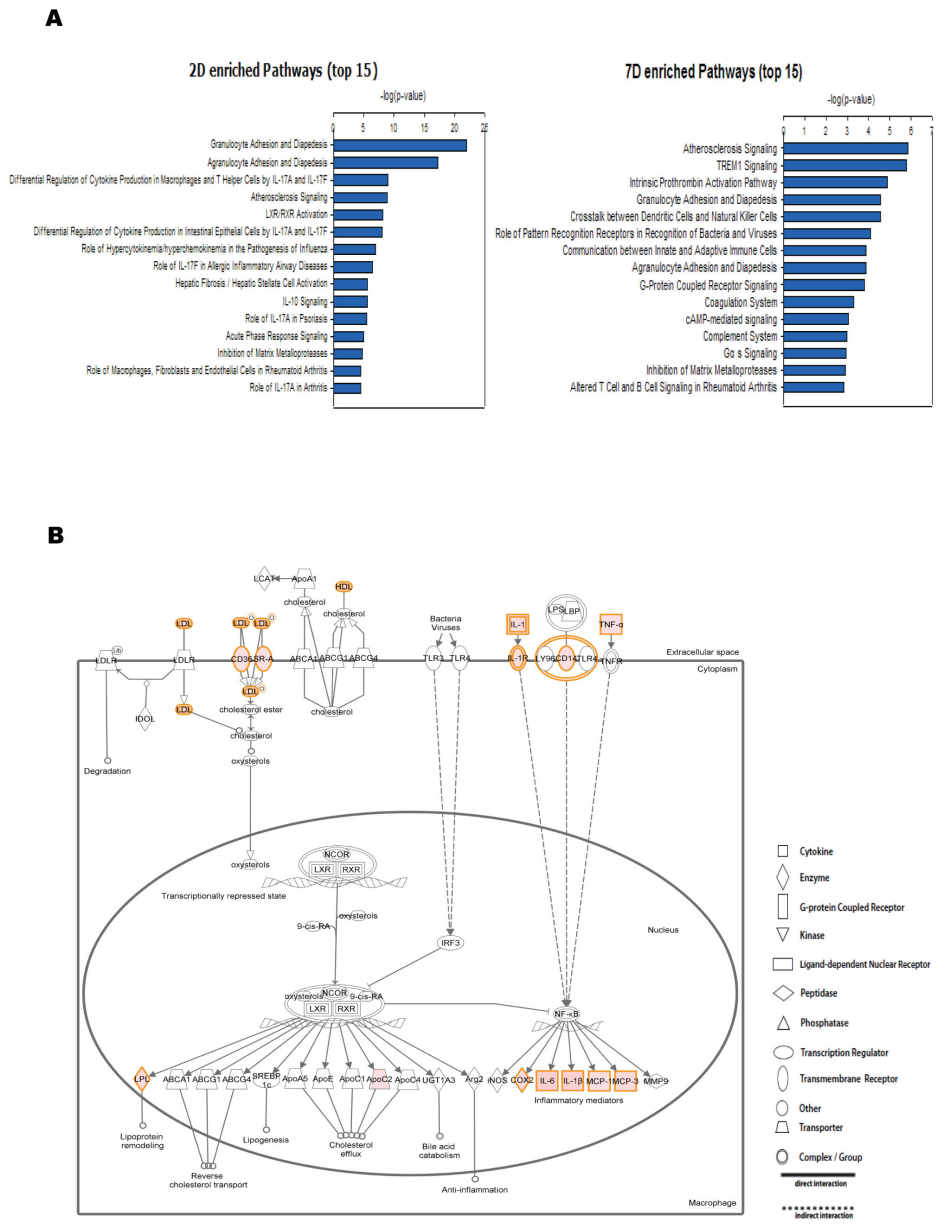
The top canonical pathways enriched in the differentially expressed genes. (A) Top 15 canonical pathways enriched in top 10% up/down-regulated genes of 2D/CTR and 7D/CTR are shown. The –log(P value) of the enrichment of each canonical pathway was plotted. (B) LXR/RXR Activation pathway up-regulated genes are colored pink. The color of a gene reflects its fold change. The higher the fold change the deeper the color.

Further, we analyzed the biological processes enriched in genes that changed most significantly (top 10% of the genes fold change > 2) in 2D and 7D by using Ingenuity Pathway Analysis (IPA). For 2D and 7D, the top 15 enriched canonical pathways are shown in Figure 2A. IPA analysis revealed that 2D and 7D shared many significantly over-represented pathways. Among the top enriched pathways (p < 0.05), 19 appeared in both 2D and 7D. These included the LXR/RXR Activation, Atherosclerosis Signaling and TREM1 Signaling pathways among others. In addition to these shared pathways, there were also different canonical pathways in 2D and 7D. For example, among the enriched pathways, Glutamate Receptor Signaling, which included mostly down-regulated genes because of increased neuron cell death, only appeared in 7D.

In 2D, the LXR/RXR pathway was one of the top enriched pathways, indicating the activation of the LXR/RXR pathway in the acute phase (Figure 2B). Among the ~50 genes that are involved in LXR/RXR pathway, 15 genes were up-regulated by more than 20-fold, including Tnf, Il6 and Il1b, which participate in multiple pathways (Table S4).

We also extended the above analysis by using more relaxed fold change criteria (>2 fold in both directions and >10 fold in both directions respectively), and identified similar critical pathways and networks such as LXR/RXR activation pathway, Atherosclerosis Signaling and others.

### Alternative splicing

**Figure 3:**
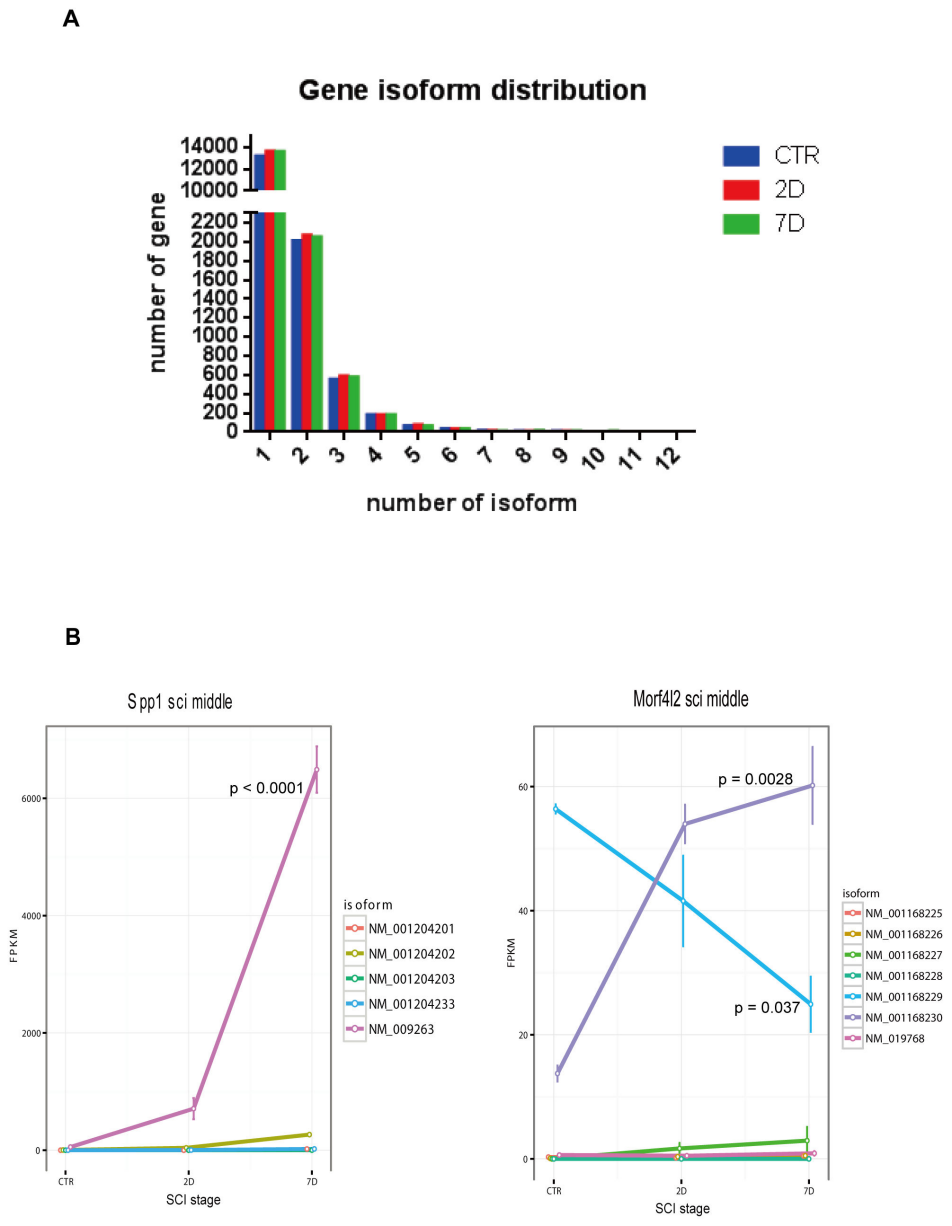
Alternative splicing analysis. (A) The annotated isoform numbers per gene (x-axis) were plotted over number of genes (y-axis). (B) Splicing isoform expression of genes Spp1 and Morfl2 in acute and subacute phases. Error bars represent ±SEM. P values for transcript NM_009263, NM_00116829 and NM_001168230 were calculated by one-way ANOVA.

An advantage of RNA-Seq technology is its ability to discern expression levels of the splicing isoforms of genes (Table S2), thus providing yet another layer of information that can be critical for future pharmacological interventions [26]. We counted the number of genes that have more than one annotated isoform expressed in all stages of SCI. There were 2741, 2946 and 2906 in CTR, 2D, 7D stages respectively. Up to 12 annotated isoforms per gene were observed, although the majority of genes (99%) had 1-5 isoforms (Figure 3A). The maximum likelihood estimation of expression level of each gene’s isoforms was performed using the cufflinks software. Among the differentially expressed genes, 10327 isoforms changed by >2 fold between at least 2 stages during SCI (CTR, 2D and 7D). The isoforms showed dynamic expression changes over the time course of the injury. We describe two interesting examples in Figure 3B.

The gene Spp1 (osteopontin, Opn) is a cytokine that is up-regulated after cell injury and tissue repair [45]. Previous studies have shown that blocking Spp1 expression at sites of cutaneous wounding resulted in reduced granulation tissue, scarring and inflammation-associated fibrosis [46]. Interestingly, Spp1 has also been shown to stimulate axon growth in retinal ganglion cells [47]. Our RNA-Seq profiling of Spp1 shows a dramatic increase in its overall expression through the acute and subacute phases of SCI, a result that is consistent with the previous studies. In addition, we found that NM_009263, one of the 5 isoforms of Spp1, is the major isoform in spinal cord tissue and is responsible for the overall increase in expression (one-way ANOVA p < 0.0001) (Figure 3B left panel). Several other isoforms are also induced compared to the CTR in 2D and 7D, but their absolute expression values are much lower than NM_009263. The distinct functions of these isoforms are still to be investigated.

Although many genes, such as Utg1a, Vegfa, and Ptprc, showed similar changing patterns in its different isoforms, there are a number of genes whose isoforms changed in the opposite directions. Gene Morf4l2 (mortality factor 4 like 2) is an example of “isoform switching” at different stages of SCI. Two of the Morf4l2 gene’s major isoforms, NM_001168229, and NM_001168230 show distinct expression patterns during acute/subacute phases (Figure 3B right panel). Isoform NM_001168229 expression level was higher in the CTR sample and it steadily declined after SCI (one-way ANOVA p = 0.037); while the NM_001168230 level was lower in the CTR sample and higher in 2D and further increased in 7D (one-way ANOVA p = 0.0028). NM_001168230 became the major isoform after injury. The function of Morf4l2 is not yet clear and thus it will be interesting to investigate the functions of its isoforms in CNS and SCI.

### Establishing the systems based analysis framework

**Figure 4:**
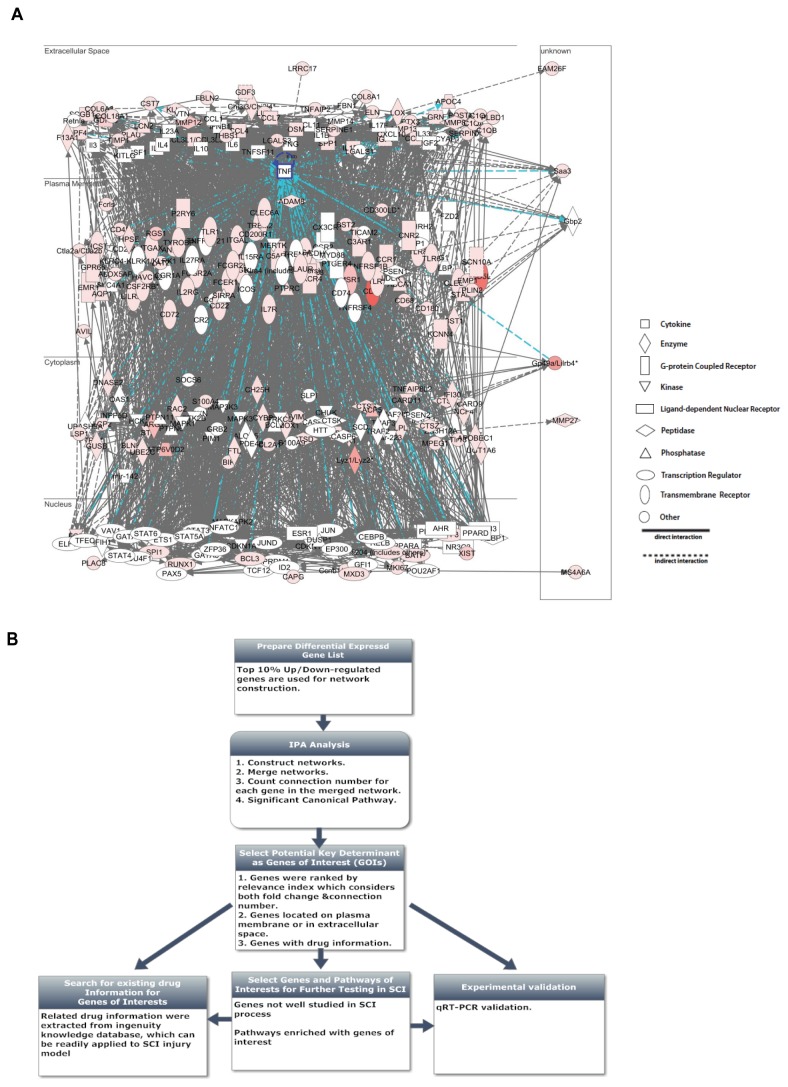
Developing a systems based analysis framework to identify key determinants in the global gene network. (A) Network constructed in 7D stage using top 10% up-regulated genes. Gene TNF (tumor necrosis factor) was highlighted with its connected edges (in blue). (B) The workflow of a systems based analysis framework in identifying potentially important genes.

To obtain a comprehensive view of the complex mechanism of SCI, we developed a systems biology based analysis framework to identify potentially important genes in the global gene network for further testing. We first constructed gene regulatory networks for 2D and 7D stages separately using IPA software and merged them into one global network for each stage (see Materials and Methods) (Figure 4A). To construct reasonable size networks that suitable for downstream analysis, top 10% up/down-regulated genes were used. After constructing networks that capture the essences of the observed transcriptomic changes in SCI, we searched for key determinants in these networks. Several criteria were used in combination to select genes of interests (Figure 4B). First, genes that are significantly differentially expressed are likely to be involved in the SCI process. Secondly, hub genes, which have a large number of interactions with other molecules (connection number in the network) usually play an important role in a network [48,49,50]. Thirdly, we are particularly interested in molecules located on the plasma membrane and extracellular space of cells because they are easily targeted by drugs [51]. These also include the neighborhood genes brought into the network by IPA analysis.

**Figure 5:**
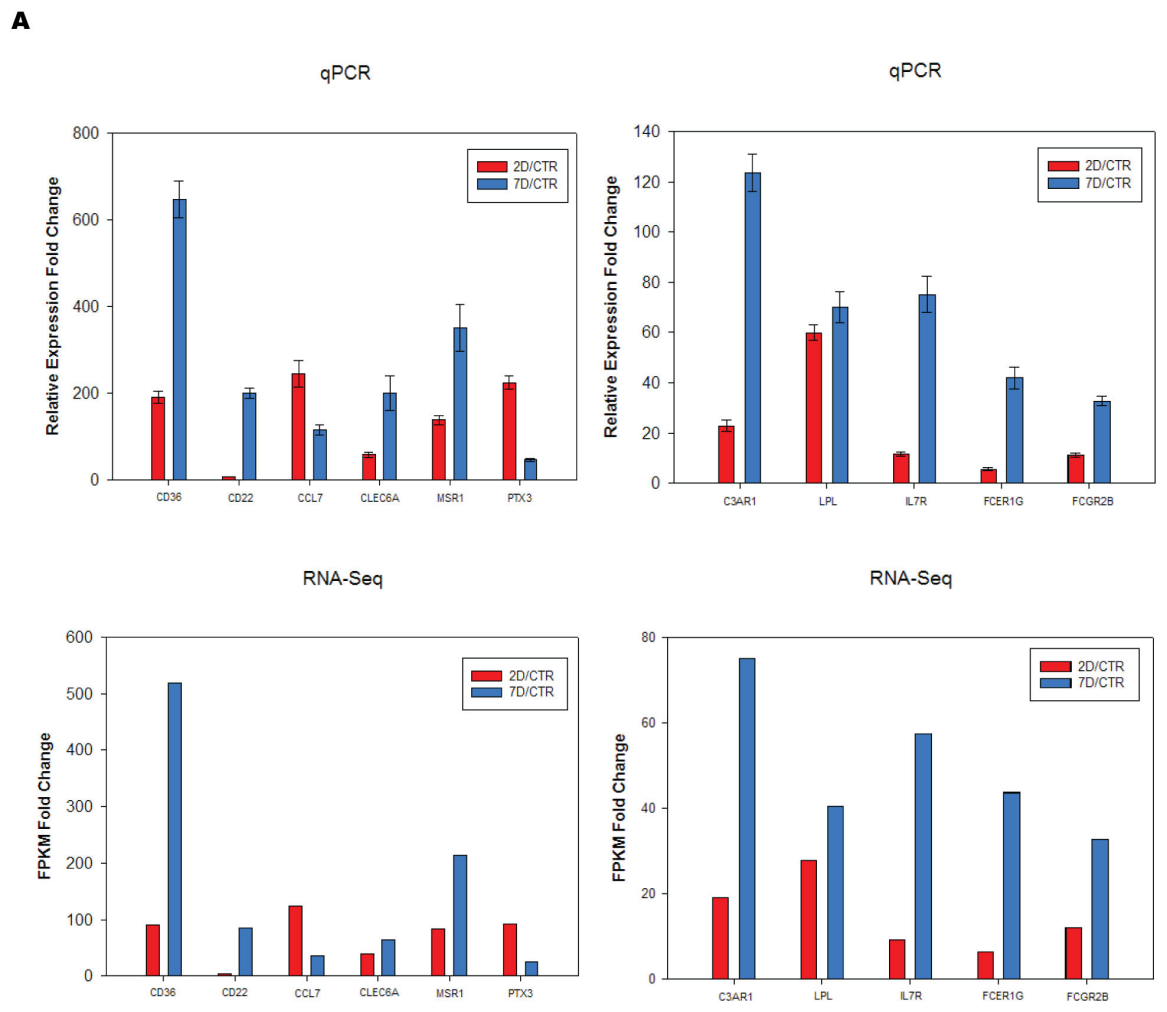
Validation using qRT-PCR. Relative expression fold change from qRT-PCR were calculated using 2^-ΔΔCt^ method. Error bars represent ±SD (n=3). FPKM fold change were the ratio of average FPKM between samples.

To better indicate the relevance of a gene, we calculated a Relevance Index (RI) number that considered both the fold change and the connection number of a gene in the constructed network and ranked the genes in that network based on this number (Table S5, Table S6 and Table S7). In the **2D** network, genes known to be involved in SCI, such as Il6, Tnf, Il1b, Ccl2 (MCP-1) were ranked at the top of the list, supporting the validity of our method [16]. We have also incorporated pharmacogenomic information from IPA into our analysis. Among the genes identified, some have existing drug information in ingenuity knowledge base can be readily tested in SCI animal models (Table S5, Table S6 and Table S7). Using the above described criteria, we identified not only well studied genes, but also genes that have not been carefully studied in SCI, including Cd36, Lpl, C3ar1 and Msr1 [52]. We performed qRT-PCR validation of the differential expression of the genes of interests (Figure 5A). The expression changes (SCI 2D/CTR and 7D/CTR) were very consistent with RNA-Seq results.

### GSEA analysis of functional gene set enrichment and the characterization of gene markers

To understand the biological implications of our global characterization, we performed a series of analysis to extract the specific functional gene sets, pathways and networks involved. Cell damage and death are the fundamental events in SCI. Understanding how they are triggered is important clinically and experimentally. Previous studies reveal that inflammation, hypoxia, nutrients supply deficiency are the main causes for the secondary spinal cord injury, and some of these genes are intensively studied. Compared with the conventional gene specific research, RNA-Seq analysis provides a genome-wide view of the systemic changes during the spinal cord injury. GSEA analysis (using the Molecular Signatures Database (MSigDB) v 3.0) [37,53], revealed hundreds of enriched gene sets among our differentially expressed genes, indicating the complex mechanism of spinal cord injury. Those gene sets related to inflammation and apoptosis ranked high among the enriched ones. Gene sets related to hypoxia [54], metabolic stress [55], glucose deprivation [56] and reactive oxygen species [57] were also enriched in both 2D (Figure S4 A) and 7D samples (Figure S4 B) [55]. IPA analysis of the leading edge genes (see methods) involved in these gene sets further indicated that these genes formed networks that function in cell death and survival (data not shown). Notably, some of the nodes in the hypoxia and nutrient gene networks are also the key factors in inflammation, such as Il6 and Tnf. When we examined the enriched gene sets for reactive oxygen species (ROS), we found that the genes can be further classified into two subsets. The gene expression of deoxygenases, such as Sod1 and Sod2 decreased in the sub-acute phase of spinal cord injury, while other ROS scavengers, such as Hmox1 and Txnrd1 increased.

**Figure 6:**
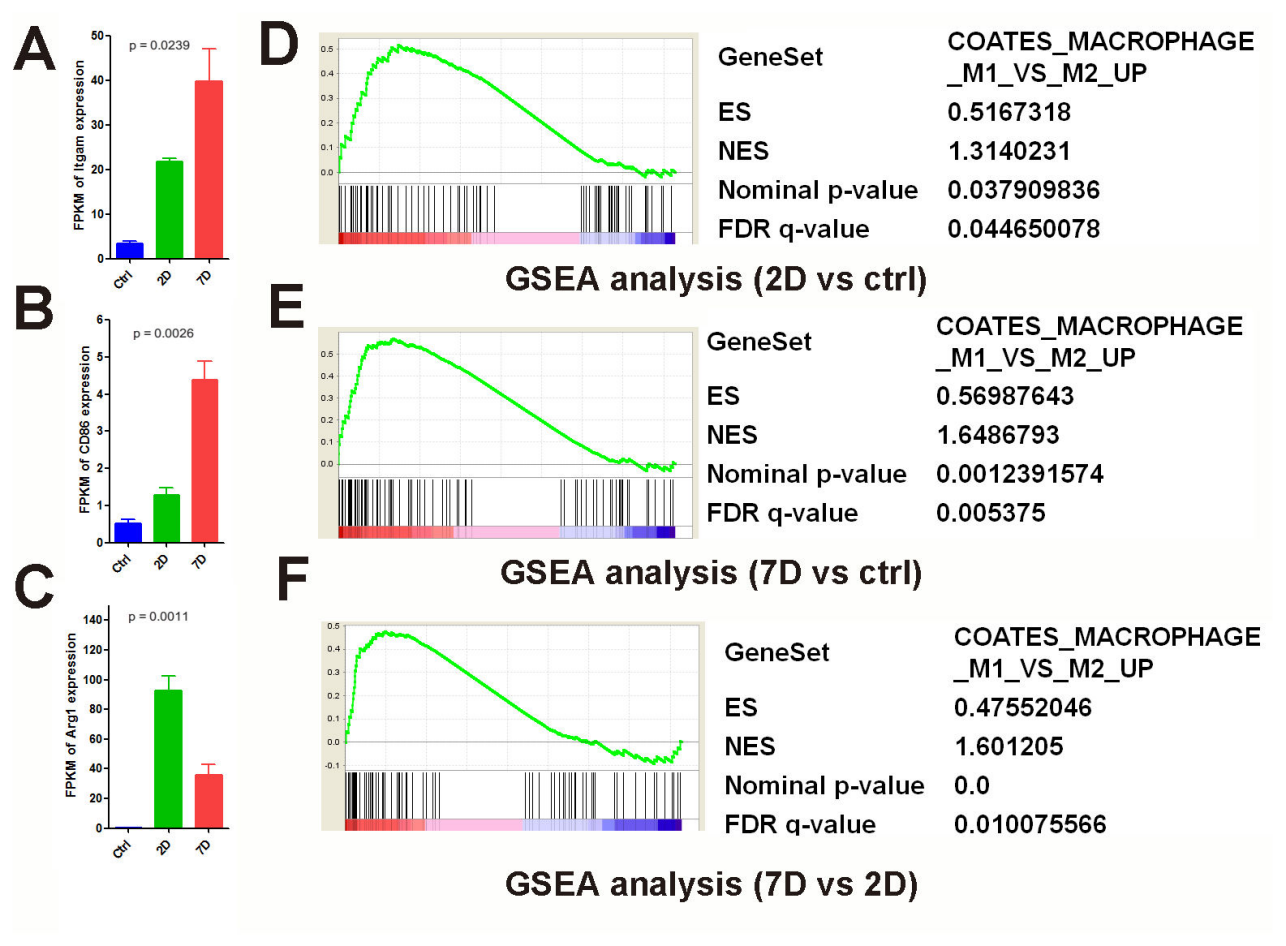
The expression of macrophage marker genes in both acute and subacute phases of SCI. Expression profile of the common macrophage marker Itgam (A), M1 specific marker CD86 (B) and M2 specific marker Arg1(C) during the time-course of SCI. P values were calculated by one-way ANOVA. GSEA analysis: differential gene expression was ranked by fold change (x-axis: 2D vs control (D), 7D vs control (E), 7D vs 2D (F)). The most up-regulated genes are shown on the left side (red), while the most up-regulated genes were shown on the right side (blue). Black bars represent the positions of the M1 vs M2 up-regulated signature genes in the ranked list. Enrichment score (ES, Y-axis) reflects the degree the genes are overrepresented. When the distribution is at random, the enrichment score is zero. Enrichment of signature genes at the top of the ranked list results in a large positive deviation of the ES from zero. NES, normalized enrichment score; FDR, false discovery rate-adjusted q value.

**Figure 7:**
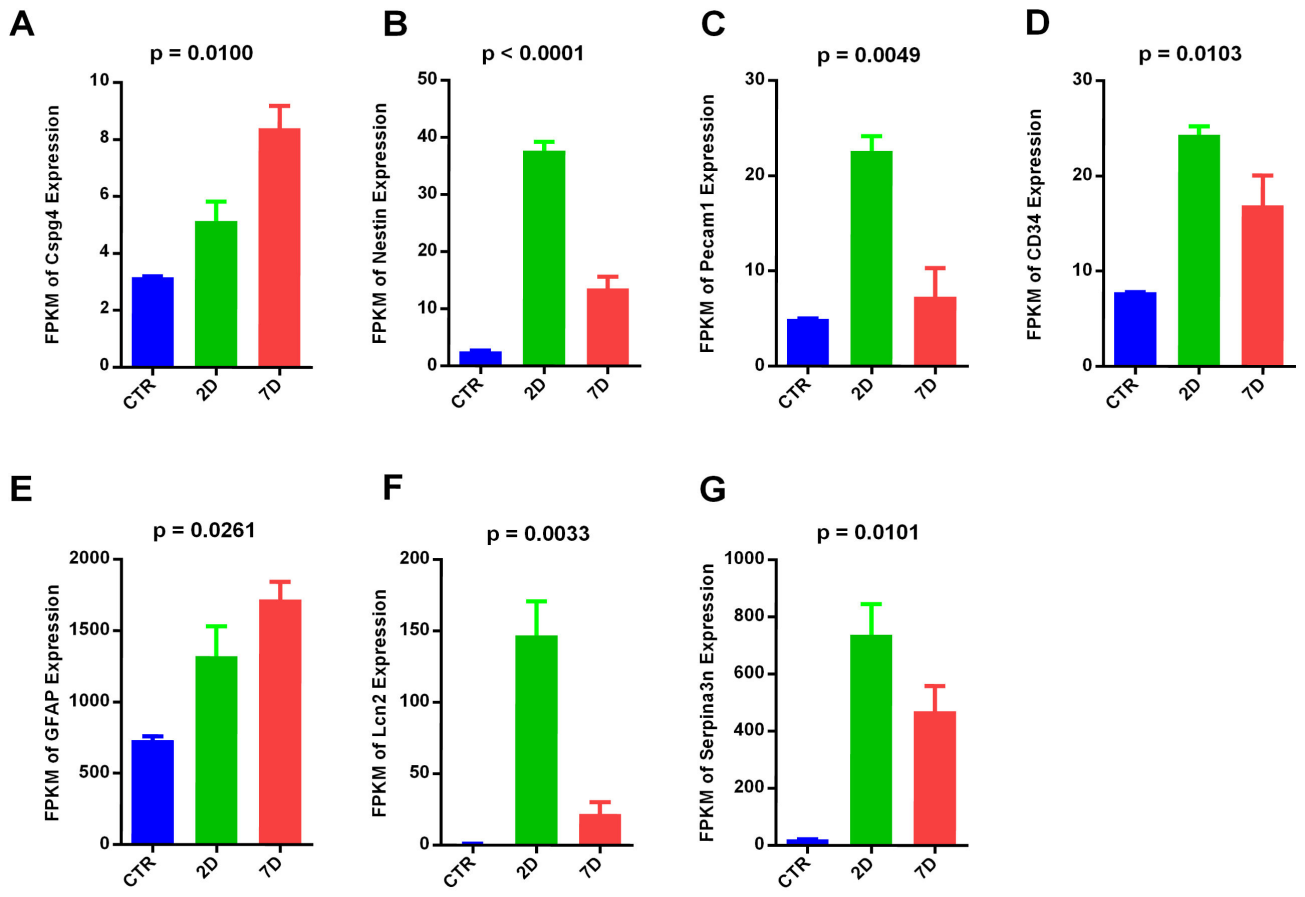
Expression dynamics of cell markers of various cell types. Expression profile of OPC marker Cspg4, also known as NG2 (A), neural stem cell marker Nestin (B), endothelial marker Pecam-1, also known as CD31 (C), hematopoietic stem cell marker CD34 (D), reactive astrocyte markers GFAP(E), Lcn2 and Serpina3n (F,G), are shown. P values were calculated by one-way ANOVA.

Complicated interactions exist between immune cells and neural cells after SCI. It is important to understand the cell types involved in this process and their interaction dynamics. Specific gene markers for macrophages (Itgam, Cd86, Arg1) (Figure 6), oligodendrocyte precursor cells (OPC: Cspg4/Ng2) (Figure 7A), neural stem cells (NSC: Nestin) (Figure 7B), endothelial cells (Pecam-1, also known as Cd31) (Figure 7C), and hematopoietic stem cells (HSC: Cd34) (Figure 7D) were selected based on the literature [58,59,60,61]. Their expression levels were compared during acute and subacute phases of SCI.

Previous studies showed that there are two subsets of macrophages which have distinct functions [59,60]. The M1 marker, Cd86, increased in 7D (one-way ANOVA p=0.0026) (Figure 6B), whereas the M2 marker, Arg1, increased in 2D (one-way ANOVA p=0.0011) (Figure 6C). Furthermore, GSEA analysis revealed that, although the M1 up-regulated gene set was enriched in both 2D and 7D samples compared with CTR(Figure 6D, E), the M1 specific gene set was further enriched in 7D samples when compared with 2D samples (Figure 6F), These data implied that M1 macrophages increased in 7D samples, while M2 macrophages decreased [60]. In the M1 up-regulated gene set, genes such as Fgd2 and Tifab etc were significantly higher in 7D samples [62,63].

We also observed dynamic changes in astroglial markers. Gfap is one of markers for reactive astrocytes after CNS injuries [64]. Its expression is up-regulated in SCI acute and subacute phases (one-way ANOVA, p = 0.0261) (Figure 7E). Lcn2 and serpina3n have been shown as markers of the early phase of reactive astrogliosis in both ischemia and neuroinflammation injury models (Figure 7F,G). They are both up-regulated in 2D and 7D and are expressed higher in 2D compared to 7D [65].

## Discussion

Although extensive work has been carried out to understand the pathophysiology of SCI, a comprehensive view of the underlying molecular mechanisms and pathways is still lacking. Limited by resource and technology, previous studies of SCI usually focus on known pathways and a small number of genes [5,6,7,8,66,67]. In this study, we systematically characterized the transcriptome of spinal cord injury at acute (2D) and subacute (7D) phases by RNA-Seq. With such an informative resource, we can now better understand the pathological processes of SCI through integrative analysis of the gene expression data.

One important advantage of massively parallel sequencing is that it is more quantitative than microarray [68]. Given its higher sensitivity, specificity and wider dynamic range, RNA-Seq can accurately measure gene transcription changes at a genome-wide scale, including known and unknown transcripts. Our study is designed to reflect the transcriptomic change of injured tissue samples which include multiple types of cells. In order to reconstruct reliable gene structures and obtain accurate expression level estimations, our procedure focused on known genes and isoforms [32]. These reference datasets are a valuable resource for the SCI research community for the derivation of new hypotheses. Importantly, RNA-Seq also enables splicing isoform identification and expression level estimation, thus providing useful information for increasing the specificity of drug design and reducing potential side effects.

Furthermore, in this study, we established a systems biology based framework to extract potential key genes from constructed networks of the SCI acute and subacute phases. Gene profiling generates a large amount of information to be mined. Recent development of network analysis made it possible to better understand the hierarchical relationships among genes involved in certain biological processes. However, currently there is no systematic method to estimate the importance of the genes in the network and to identify the potential key determinants for further experimentation. The analysis framework that we have developed combines existing knowledge from the literature, such as molecular interactions and cellular localization information, with our differential expression data. Based on these criteria, we were able to prioritize genes of interest that exhibited specific characteristics, thus demonstrating the validity of our approach.

First, this list contains many important genes known to be involved in SCI. In addition to Tnf, Il6, Il1b which were well studied in SCI, many other genes, such as Hmox1 [69], Ccl11 [70], and Ccr1 [71], also gained attention. Hmox1 is an inducible cytoprotective enzyme and has anti-inflammatory and antioxidant effects after spinal cord injury [69]. Expression of Ccl11 is associated with immune response modulation and protection against neuroinflammation [70]. Ccr1 was suggested to have a crucial role in the chronic central pain mechanisms after SCI [71]. Very recently, genes such as Lcn2 [72] and Tlr4 [73] were also found to play important roles in SCI. Lcn2 (Lipocalin 2) is important in the defense against bacterial infection by interfering with bacterial iron acquisition. It has also been shown that a lack of Lcn2 reduces secondary damage and improves locomotor recovery after spinal cord injury [72]. Tlr4 expression was found to lead to greater synaptic loss, and was correlated with increased astrogliosis and up-regulation of pro-inflammatory interleukins [73].

Secondly, most genes identified by our method are involved in relevant biological processes (such as ‘inflammation’ or ‘nerve system development’ functional categories) and pathways in SCI. For example, among pathways that are enriched in the 2D network, Ccl2, Ccl7, Msr1, Il1rn, Il6, Il1b, Tnf, Ptgs2, Lpl and Cd36 are involved in ‘LXR/RXR Activation’ (p = 1.38E-09, Fisher Exact Test for pathway enrichment), and Il1rn, Osm, Cfb, Il6, Il1b, Tnf, Serpine1 and Hmox1 are involved in ‘Acute Phase Response Signaling’ (p=8.58E-6, Fisher Exact Test). The LXR/RXR pathway is one of the top enriched pathways in 2D and is also significantly enriched in 7D. The LXR/RXR pathway is activated by oxysterols and intermediates in the cholesterol synthetic pathway, which are important for the metabolic conversion of cholesterol to bile acids in the liver [74]. In addition, the LXR pathway also plays an important regulatory role in several metabolic signaling pathways in adipose tissue, such as glucose uptake and de novo fatty acid synthesis [74]. However, whether this pathway has similar functions as in the nervous system remains largely unknown. Previously, multiple studies have revealed that some genes of this pathway, such as Lpl, are widely expressed in multiple cell types in the spinal cord and are considered to be essential in regulating feeding behavior and maintaining normal neuronal function [75,76], further supporting a role for the LXR pathway in the nervous system. In addition to lipid metabolism, the LXR/RXR pathway was also found to be essential for the endogenous anti-inflammatory response in macrophages [77]. It will be intriguing to investigate the roles of other genes in this pathway in SCI.

Among pathways that are enriched in the 7D network, Ccl2(MCP-1), Mmp3, Cxcr4 and Ccr2 genes are involved in the ‘Atherosclerosis Signaling’ pathway (p=1.03E-5 Fisher Exact Test), and Cd28, Osm, Lgals3 and Clec7a genes are involved in the ‘Role of Pattern Recognition Receptors in Recognition of Bacteria and Viruses’(p=4.14E-4 Fisher Exact Test). Notably, the ‘Atherosclerosis Signaling’ pathway is the top enriched pathway in 7D; Ccl2(MCP-1), Cxcr4 and Ccr2in this pathway have been shown to play a role in SCI. Ccl2 chemokine and its receptor Ccr2 contribute to neuropathic pain development after spinal cord injury [78]. Cxcr4 are key regulator of neuro-repair processes after brain ischemia and spinal cord injury; inhibiting Cxcr4 can enhance re-myelination and improve recovery [79,80]. C3ar and C5ar in the ‘Role of Pattern Recognition Receptors in Recognition of Bacteria and Viruses’ pathway play a role in the inhibition of apoptosis. Genes related to ‘nerve system development’ included mostly down-regulated genes because of increased neural cell death. Glutamate Receptor Signaling pathway was enriched.

Importantly, based on this valid approach that we developed, we were able to identify new genes of interests for further investigation. The qRT-PCR validation indicates a highly consistent expression pattern with the RNA-Seq result. Among these, some genes have been indicated to have a role in other pathological conditions, but not in SCI. For example, Lpl can modify the synaptic loss and remodeling processes in a brain injury model [76] and has a potential role in Alzheimer’s disease [81]. It may also play a role in the pathophysiological response to cerebral ischemia reperfusion [82]. Cd36 inhibition was suggested as a viable strategy to enhance possible recovery in stroke [83]. Ptx3 was found to have protective role in seizure-induced neurodegeneration [84] and its expression increases in the spinal cord during EAE (experimental autoimmune encephalomyelitis) [52]. Therefore, we demonstrated an example of how global gene profiling can be translated into identifying genes of interest as potential therapeutic targets for functional tests. We have also incorporated pharmacogenomic information into our analysis. Among the genes identified, the ones with existing drug information in IPA database can be readily tested in SCI animal models.

Additionally, by analyzing the transcriptome signatures in our data using GSEA, we identified a large number of gene sets that are significantly enriched in the SCI process. Conventional studies examine genes of interest individually; transcriptomic studies provide the expression of the gene markers simultaneously and thus are more informative. Therefore, with cell type specific markers, we can examine the responses of different cell types and subtypes of cells in the spinal cord injury environment and during the injury time course. Such information provides valuable clues for detailed biological study in the future. For example, previous studies revealed that there are two subsets of macrophages, M1 and M2. M1 can be identified as Cd86+, and M2 are Arg1+ [59,60]. We found that M1 macrophages increased in 7D samples, while M2 macrophages decreased. M1macrophages have been shown to be harmful for regeneration, whereas M2 macrophages are potentially beneficial for regeneration [60]. This suggests that the reduction of the M1/M2 ratio in the subacute phase and the modulation of M1 properties may potentially promote regeneration. In the M1 up-regulated gene set, genes such as Fgd2 and Tifab etc are significantly higher in 7D samples. Fgd2 was found to play a role in vesicle trafficking in immune cells [62] and Tifab may be an important regulator during the maturation of macrophages [63]. It is possible that these genes may play a role in distinguishing the functions of M1and M2 macrophages. This could be tested by further experimentation.

A recent transcriptome study characterized the induced genes [65] of reactive astrogliosis in two different injury models (ischemia and neuroinflammation). We compared the top 50 induced genes from these two injury models with our RNA-Seq dataset and found most induced genes in both injury models are up-regulated in our SCI 2D and 7D data. Thereby, ischemia and neuroinflammation both contribute to the activation of reactive astrocytes and gliosis in SCI acute/subacute phases.

In summary, our study has not only characterized the dynamic change of genes known to play a role in SCI, but also provided new potential key determinants and pathways in this pathological process. The examples that we have described here are only a brief demonstration of the potential value of this resource, and from our study there is a large amount of biological information available to be explored by the scientific community. By following up the hypotheses generated from these analyses, we hope to identity new mechanisms and therapeutic targets for reducing secondary tissue damage and promoting regeneration.

## Supporting Information

Table S1 **Summary of read mapping.**
(TIF)

Table S2 **Expression value (FPKM) of genes and isoforms.**
(XLSX)

Table S3 **Top enriched functional categories in acute and subacute SCI.**
(XLSX)

Table S4 **The expression of genes involved in LXR/RXR pathway 2 days after SCI.**
Abbreviations: E (Extracellular Space); P (Plasma Membrane); C (Cytoplasm); N (Nucleus); Y (there is drug for the gene in IPA database).
(XLS)

Table S5 **Genes of interest identified from network constructed in acute SCI(2D).**
Genes were listed in the order of their Relevance Index number. RI = abs(log(Fold change)) *(Connection number).
(XLSX)

Table S6 **Genes of interest identified from network constructed with up-regulated genes in subacute SCI (7D).**
Genes were listed in the order of their Relevance Index number. RI = abs(log(Fold change)) *(Connection number).
(XLSX)

Table S7 **Genes of interest identified from network constructed with down-regulated genes in subacute SCI (7D).**
Genes were listed in the order of their Relevance Index number. RI = abs(log(Fold change)) *(Connection number).
(XLSX)

Figure S1 **Histological staining of the injured spinal cord by the iron-eriochrome cyanine R (EC) staining shows a moderate contusion injury.**
The contusion SCI resulted in a central injury devoid of neural tissue and an outer rim of spared white matter in the injury epicenter at both 2 and 7 days after contusion. The central injury gradually decreased while the peripheral spared white matter gradually increased caudally and rostrally away from the epicenter center. The injury was further increased in 7D in comparison to 2D after SCI.
(TIF)

Figure S2 **Sample correlation and clustering analyses.**
(A) Pearson correlation coefficients between samples. (B) Unsupervised hierarchical clustering of CTR, 2D, and 7D RNA-Seq data (top 3000 genes with highest variation across SCI stages were used). (C) Clustering of differentially expressed genes. 7239 genes whose expression changed > 2 folds were clustered into 9 groups using c-means clustering algorithm.
(TIF)

Figure S3 **Comparison of RNA-Seq and microarray data.**
(A) Spearman correlation between RNA-Seq data and microarray data. Only genes that are detected by both platforms (15153 genes) were used (‘m’ stands for microarray, ‘r’ stands for RNA-Seq). (B) The comparison of differential expression detection. The number of DE genes (Y-axis) was plotted as a function of fold threshold used (7D/CTR, X-axis, log scale). At the same fold threshold, RNA-Seq can detect more DE genes than microarray. (C) The comparison of dynamic range. The fraction of genes (Y-axis) was plotted as a function of fold changes (7D/CTR, X-axis, log scale) detected by RNA-Seq and microarray. RNA-Seq shows a broader dynamic range for the fold change detection in SCI data. Only genes that are detected by both platforms were used.
(TIF)

Figure S4 **Analyses of the functional gene set enrichment by GSEA.**
(A) Differential gene expression was ranked by fold change (2D vs control, x-axis). The most up-regulated genes are shown on the left side (red), while the most down-regulated genes were shown on the right side (blue). Black bars represent the positions of the individual genes of the signature gene set (hypoxia signature (a), rapamycin response up-regulated gene signature (rapamycin inhibits mTOR pathway and represents the metabolic stress) (b), glucose deprivation up-regulated gene sets (c) and reactive oxygen species related gene sets (d)) in the ranked list. Enrichment score (ES, Y-axis) reflects the degree the genes are overrepresented. When the distribution is at random, the enrichment score is zero. Enrichment of signature genes at the top of the ranked list results in a large positive deviation of the ES from zero. (B) Genes were ranked according to the expression ratio (7D vs control) and further analyzed by GSEA with the same molecular signature gene sets as above and indicated as a’, b’, c’ and d’ correspondingly. ES, enrichment score; NES, normalized enrichment score; FDR, false discovery rate-adjusted q value.
(TIF)
