# Supplementary figures and images for: RNA-Seq Characterization of Spinal Cord Injury Transcriptome in Acute/Subacute Phases: A Resource for Understanding the Pathology at the Systems Level
Source: PLoS One. 2013 Aug 9;8(8):e72567. doi: 10.1371/journal.pone.0072567 (PMC3739761; doi:10.1371/journal.pone.0072567)

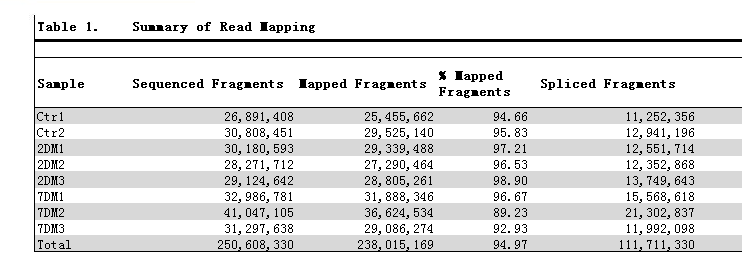

Supplement: Table S1 — Summary of read mapping. (TIF) [file pone.0072567.s001.tif]

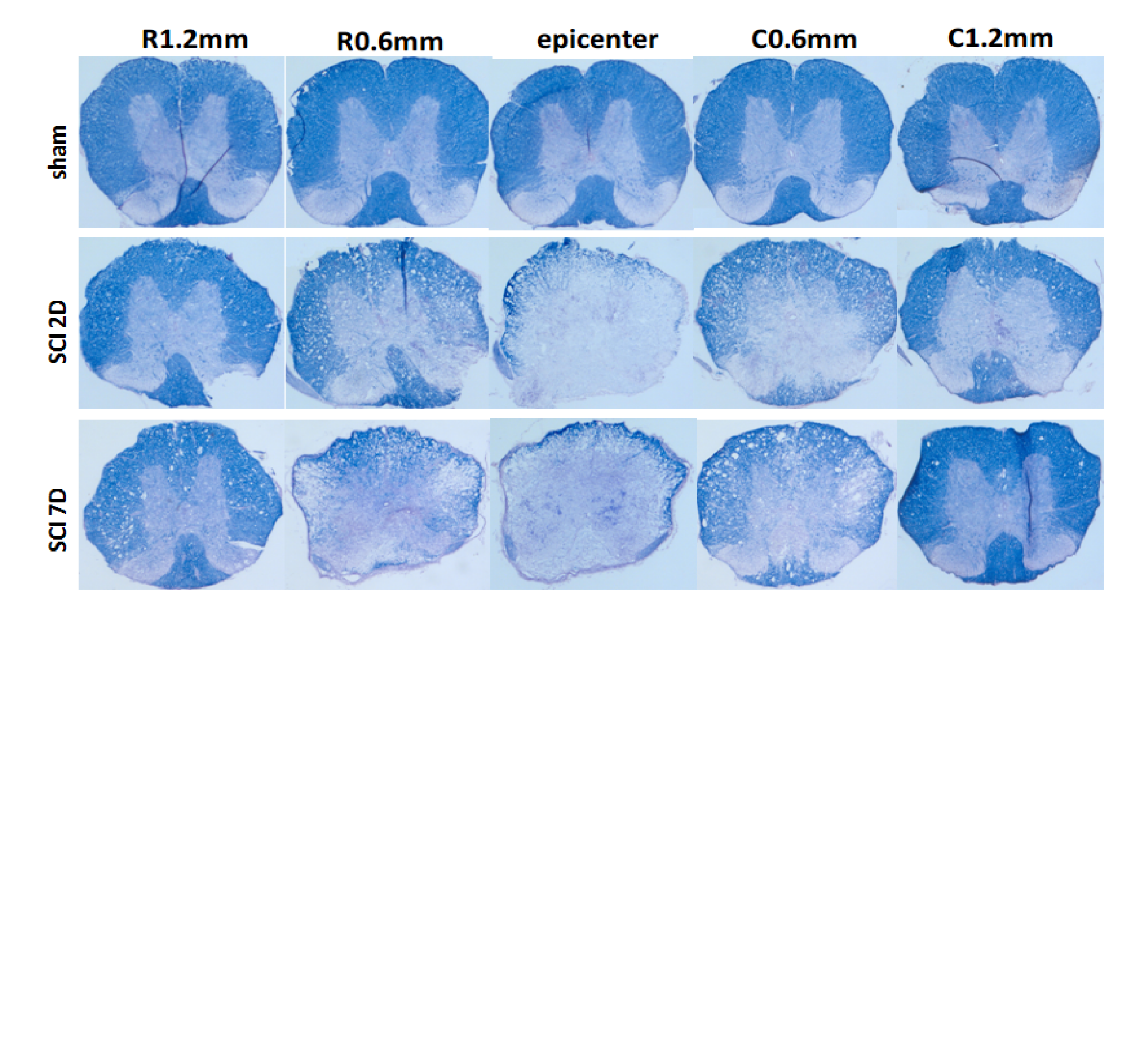

Supplement: Figure S1 — Histological staining of the injured spinal cord by the iron-eriochrome cyanine R (EC) staining shows a moderate contusion injury. The contusion SCI resulted in a central injury devoid of neural tissue and an outer rim of spared white matter in the injury epicenter at both 2 and 7 days after contusion. The central injury gradually decreased while the peripheral spared white matter gradually increased caudally and rostrally away from the epicenter center. The injury was further increased in 7D in comparison to 2D after SCI. (TIF) [file pone.0072567.s008.tif]

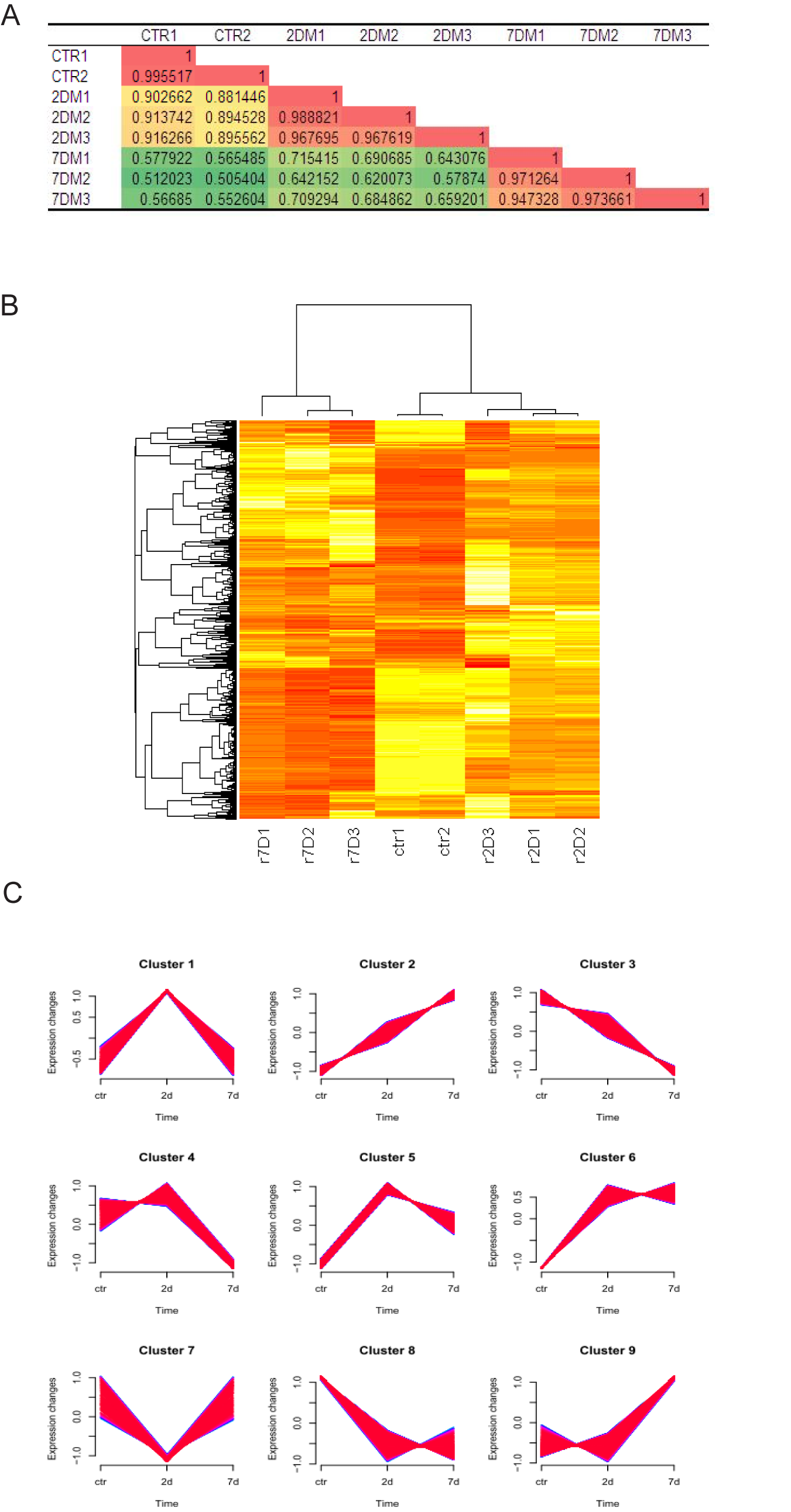

Supplement: Figure S2 — Sample correlation and clustering analyses. (A) Pearson correlation coefficients between samples. (B) Unsupervised hierarchical clustering of CTR, 2D, and 7D RNA-Seq data (top 3000 genes with highest variation across SCI stages were used). (C) Clustering of differentially expressed genes. 7239 genes whose expression changed > 2 folds were clustered into 9 groups using c-means clustering algorithm. (TIF) [file pone.0072567.s009.tif]

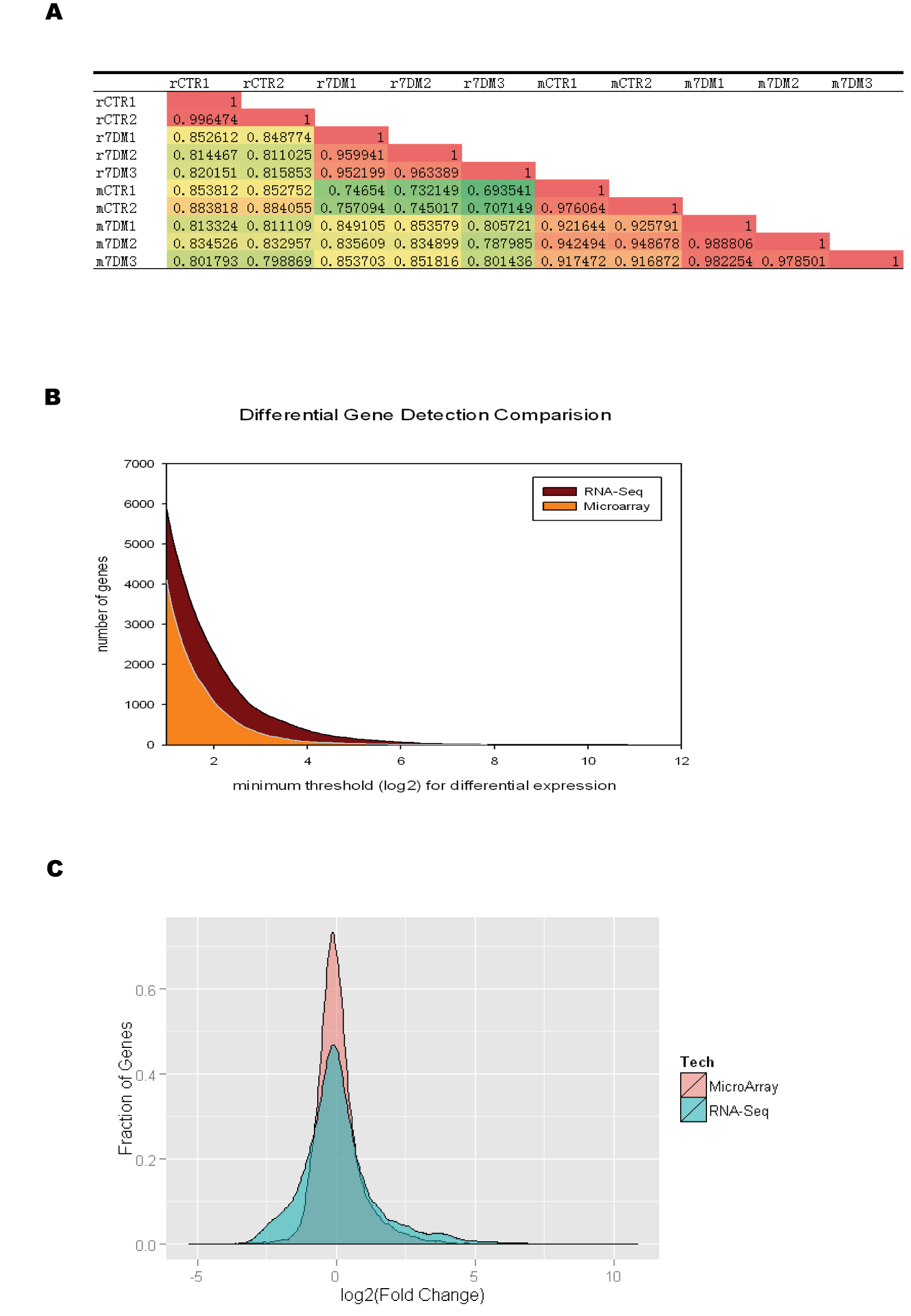

Supplement: Figure S3 — Comparison of RNA-Seq and microarray data. (A) Spearman correlation between RNA-Seq data and microarray data. Only genes that are detected by both platforms (15153 genes) were used (‘m’ stands for microarray, ‘r’ stands for RNA-Seq). (B) The comparison of differential expression detection. The number of DE genes (Y-axis) was plotted as a function of fold threshold used (7D/CTR, X-axis, log scale). At the same fold threshold, RNA-Seq can detect more DE genes than microarray. (C) The comparison of dynamic range. The fraction of genes (Y-axis) was plotted as a function of fold changes (7D/CTR, X-axis, log scale) detected by RNA-Seq and microarray. RNA-Seq shows a broader dynamic range for the fold change detection in SCI data. Only genes that are detected by both platforms were used. (TIF) [file pone.0072567.s010.tif]

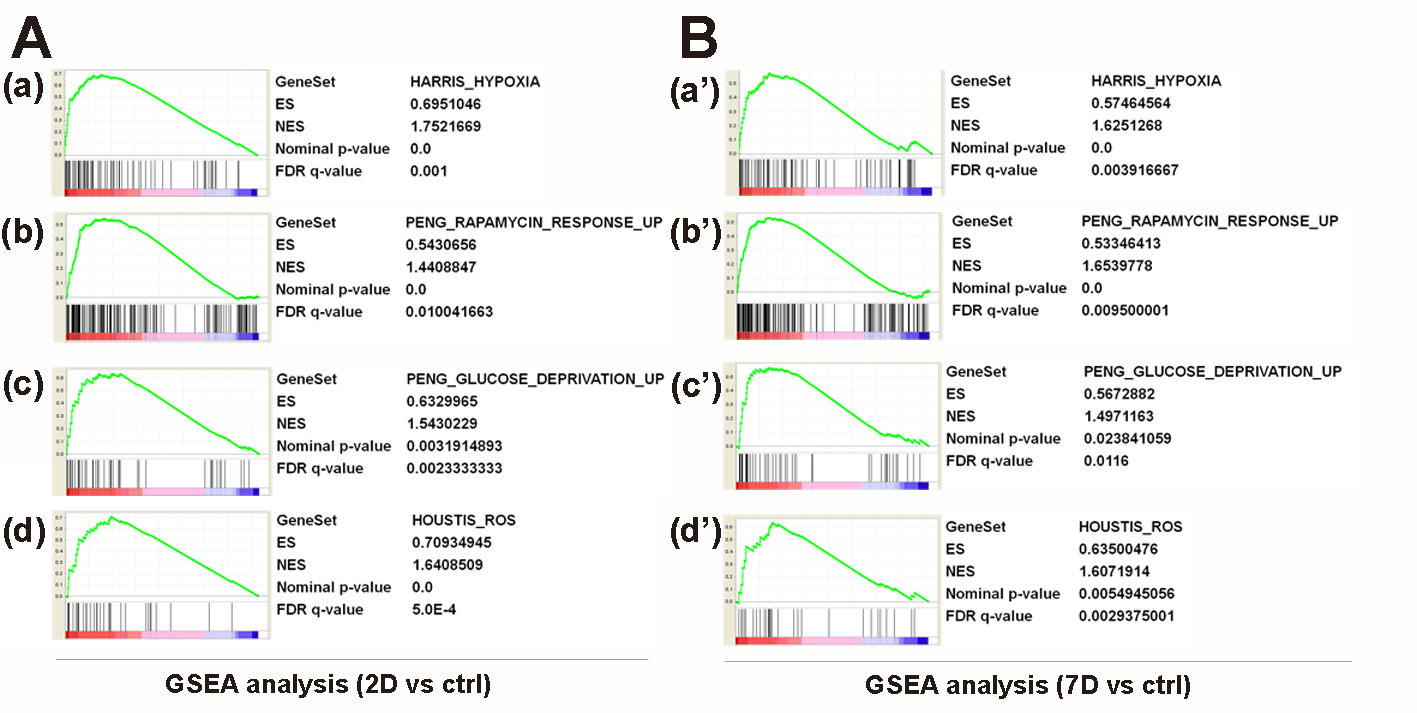

Supplement: Figure S4 — Analyses of the functional gene set enrichment by GSEA. (A) Differential gene expression was ranked by fold change (2D vs control, x-axis). The most up-regulated genes are shown on the left side (red), while the most down-regulated genes were shown on the right side (blue). Black bars represent the positions of the individual genes of the signature gene set (hypoxia signature (a), rapamycin response up-regulated gene signature (rapamycin inhibits mTOR pathway and represents the metabolic stress) (b), glucose deprivation up-regulated gene sets (c) and reactive oxygen species related gene sets (d)) in the ranked list. Enrichment score (ES, Y-axis) reflects the degree the genes are overrepresented. When the distribution is at random, the enrichment score is zero. Enrichment of signature genes at the top of the ranked list results in a large positive deviation of the ES from zero. (B) Genes were ranked according to the expression ratio (7D vs control) and further analyzed by GSEA with the same molecular signature gene sets as above and indicated as a’, b’, c’ and d’ correspondingly. ES, enrichment score; NES, normalized enrichment score; FDR, false discovery rate-adjusted q value. (TIF) [file pone.0072567.s011.tif]
